# Role of endothelial cells and angiotensin converting enzyme-II in COVID-19 and brain damages post-infection

**DOI:** 10.3389/fneur.2023.1210194

**Published:** 2023-06-30

**Authors:** Riffat Mehboob, Jens Peter von Kries, Kashifa Ehsan, Majid Almansouri, Ahmed K. Bamaga

**Affiliations:** ^1^Lahore Medical Research Center and LMRC Laboratories, LLP, Lahore, Pakistan; ^2^Screening Unit, Leibniz-Research Institute of Molecular Pharmacology (FMP), Berlin, Germany; ^3^Department of Clinical Biochemistry, Faculty of Medicine, King Abdulaziz University, Jeddah, Saudi Arabia; ^4^Neurology Division, Pediatric Department, Faculty of Medicine, King Abdulaziz University Hospital, King Abdulaziz University, Jeddah, Saudi Arabia; ^5^Pediatric Department, King Faisal Specialist Hospital and Research Center, Jeddah, Saudi Arabia

**Keywords:** endothelial dysfunction, coronavirus - COVID-19, angiotension converting enzyme, neutral Endopeptidase (NEP), substance P, SP, inflammation, vasculature

## Abstract

Severe acute respiratory syndrome coronavirus 2 (SARS CoV-2) causes coronavirus disease 2019 (COVID-19), which became a pandemic in late 2019 and early 2020. Apart from many other symptoms of this infection, such as loss of smell and taste, rashes, body aches, fatigue, and psychological and cardiac symptoms, it also causes vasodilation in response to inflammation via nitric oxide release. SARS CoV-2 affects microcirculation, resulting in the swelling and damage of endothelial cells, micro thrombosis, constriction of capillaries, and damage to pericytes that are vital for the integrity of capillaries, angiogenesis, and the healing process. Cytokine storming has been associated with COVID-19 illness. Capillary damage and congestion may cause limited diffusion exchange of oxygen in the lungs and hence hypoxemia and tissue hypoxia occur. This perspective study will explore the involvement of capillary damage and inflammation by their interference with blood and tissue oxygenation as well as brain function in the persistent symptoms and severity of COVID-19. The overall effects of capillary damage due to COVID-19, microvascular damage, and hypoxia in vital organs are also discussed in this perspective. Once initiated, this vicious cycle causes inflammation due to hypoxia, resulting in limited capillary function, which in turn causes inflammation and tissue damage. Low oxygen levels and high cytokines in brain tissue may lead to brain damage. The after-effects may be in the form of psychological symptoms such as mood changes, anxiety, depression, and many others that need to be investigated.

## Introduction

The novel SARS-CoV-2 causes an acute respiratory illness; the virus enters via the orofacial region’s mucous membranes, travels to the trigeminal ganglion, and then takes control of its peptides, including Substance P (SP). Associated with nociception and inflammation in response to noxious stimuli, SP is the primary neuropeptide, neuromodulator, and neuro-hormone of the trigeminal ganglion (TG). When SP is released, it affects blood vessels and immunological cells, causing them to secrete inflammatory mediators. In complex situations, cytokine storming starts and results in respiratory distress, bronchoconstriction, and mortality (1). Glucocorticoids and Neurokinin-1 Receptor (NK-1R) antagonists may be used to treat and relieve inflammatory symptoms. The primary offender that seems to be responsible for activating inflammatory pathways during SARS-CoV-2 infection may be SP, as discussed in our previous study (2). Neutral endopeptidase (NEP) degrades SP under normal physiological conditions while NK-1R is the receptor of SP that initiates its responses upon binding. Glucocorticoids such as dexamethasone will affect NEP and NK-1R antagonists will block the NK-1R in treatment strategy, as was shown in a previous clinical trial in patients with COVID-19 (2, 3). Numerous significant physiological and pathological functions are controlled by SP, and SP has a direct relationship with the cardiorespiratory rhythm, sleep–wake cycle, nociception, and ventilatory responses (2, 4). To cure organ damage brought on by COVID-19-driven inflammatory reactions, SP over-secretion should be stopped with NK-1R antagonists (2).

During acute lung injury, such as in COVID-19 infection, there is cellular inflammation, which is accompanied by micro thrombosis, hemorrhage along with intravascular blood coagulation. The concept of STORM-2, as proposed by (5), is the ability to implement a special pharmacotherapy strategy for COVID-19 to normalize the endothelium, manage blood coagulation, transcellular transfusion, and maintain blood pressure (5).

### COVID-19 pathogenesis in the respiratory tract

COVID-19 can be asymptomatic or have diverse manifestations ranging from mild to severe. Initially, the coronavirus-2 enters the alveolar cells of the lungs by penetrating through the transmembrane ACE-2 receptors. It leads to cytokine storming and activation of immune cells, causing respiratory distress syndrome. Inflammatory mediators are secreted in large amounts, causing organ damage and respiratory failure. Alveolar cells are damaged with microangiopathy in COVID-19 infection, causing bilateral pneumonia. Some patients develop hypercoagulable syndrome and thrombosis while damaging other organs such as the heart, kidneys, and liver, as well as the endocrine and immune systems (6). Clinical symptoms during different stages of COVID-19 may include viral infection and cytokine storm, damage to the vascular endothelium of the heart, brain, and other systems, coagulation and thrombosis in organs, and neurological problems.

The patient’s eyes, mouth, and nose are all entry points for the SARS-CoV-2 virus into their respiratory system. It may also go via its branches, V1, V2, and V3, to reach the trigeminal ganglion. The respiratory control center of the brain is the TG, which also produces significant neurotransmitters such as SP. After being activated by a nociceptive stimulus, such as a virus, SP modifies the inflammation and initiates cytokine storming. To inhibit cytokine storming, SP and its receptor NK-1R should be blocked. The main pathogenesis during COVID-19 infection includes damage to the alveolar area, which induces mild to severe clinical respiratory symptoms. Interestingly, the drugs that block Angiotensin II receptor and ACE inhibitors are frequently used in patients with COVID-19 and the patients treated with these drugs have shown increased expression of ACE-2 (7).

### COVID-19 pathogenesis in the brain

SARS-CoV-2 infects the brain through the olfactory bulbar zone. Axonal transport along the olfactory nerve, which may reach the temporal lobe and the olfactory area of the cerebral cortex, can result in brain infection. Trans-synaptic transmissions allow the virus to reach the brain stem and thalamus. The virus produces acute respiratory problems in the respiratory tract (8).

The second, more typical method is known as the “hematogenous route,” which involves blood–brain barrier (BBB) breaching and vascular endothelium destruction brought on by a coronavirus. The virus may damage the capillary endothelium by interacting with the ACE-2 protein, causing endotheliitis, which makes it easier for the virus to enter the brain. ACE-2 downregulation and increased activity of cathepsin L and transmembrane protease serine 2 (TMPRSS2) may lead to increased expression of pro-inflammatory mediators that trigger blood barrier disruption and neuro-inflammatory responses (8). Also, dysregulation of neurotransmitter signaling and hormones are important elements in the neuropathogenesis of SARS-CoV-2 infection. The RNA of coronavirus also interacts with or activates the molecular signaling pathways controlled by cell suicide molecules, pattern recognition receptors, and complement cascades thus, affecting central nervous system functions by humoral and neural pathways (9). Patients infected with COVID-19 report many neurological symptoms during and afterward, such as headaches. SARS-CoV2 may have the ability to enter and infect the human nervous system based on the intense expression and localization of the ACE-2 receptor having wide distribution in the brain. Due to the possibility of entry of coronavirus into the brain, there is strong speculation of harmful neurological effects after SARS-CoV-2 infection (10). In one of our previous perspective studies, we discussed the same threat of neurological symptoms and the possible theory of latency of coronavirus. It may become active anytime in the future, even when the patient is completely recovered (11). Another study discussed the enhanced ACE-2 levels leading to cardiovascular and neurological disorders associated with inflammatory effects. It causes nervous system damage leading to cognitive dysfunction, insulin sensitivity reduction, anxiety, depression, and behavioral disorders (12).

SARS-CoV-2 infection may also cause hemorrhagic stroke, cognitive impairments, brain fog, polyneuropathies, insomnia, and short-term memory loss. Although there are scattered information and reports, there is still a lack of concrete evidence and thus, further studies are needed (13).

### Role of endothelial cells

The lungs’ metabolic or transforming function postulates that when venous blood changes to arterial blood, biochemical components, including adrenaline, nitric oxide, angiotensin I and II, bradykinin, endothelin, and prostaglandins, are actively synthesized or degraded. As a result, the lungs work as a filter to determine the regulatory makeup of the hemi-dynamic system’s biochemical components (14). In many organs, including the lungs, the endothelium of blood arteries is an endocrine tree. Coronavirus-2 targets several significant pathophysiological processes that are focused on one area. The ACE-2 enzyme is the primary cellular target of viral aggressiveness. The normal production of angiotensin and bradykinins by ACE/ACE-2 is inhibited by coronavirus, which throws off the balance of the blood vessels. The pathophysiology and molecular features of COVID-19 must be understood.

Endothelial cells, smooth muscles, and pericytes organize themselves to form blood vessels in a biochemical and physical environment indicated by the term vascular niche. The vascular system has a complex and intricate process depending upon many intrinsic and extrinsic responses (15). In some mutational studies of mice and fish, the vascular system was observed to be highly sensitive to genetic disruption and had prospective targets for therapeutic interference (16). After the formation of blood vessels, molecules are released that are involved in the recruitment of endothelial progenitor cells, hematopoietic stem cells, and mesenchymal stem cells. The location of endothelium cells in proximity to newly developing cellular elements indicates their importance in the maturation of organs (17, 18).

**Figure 1:**
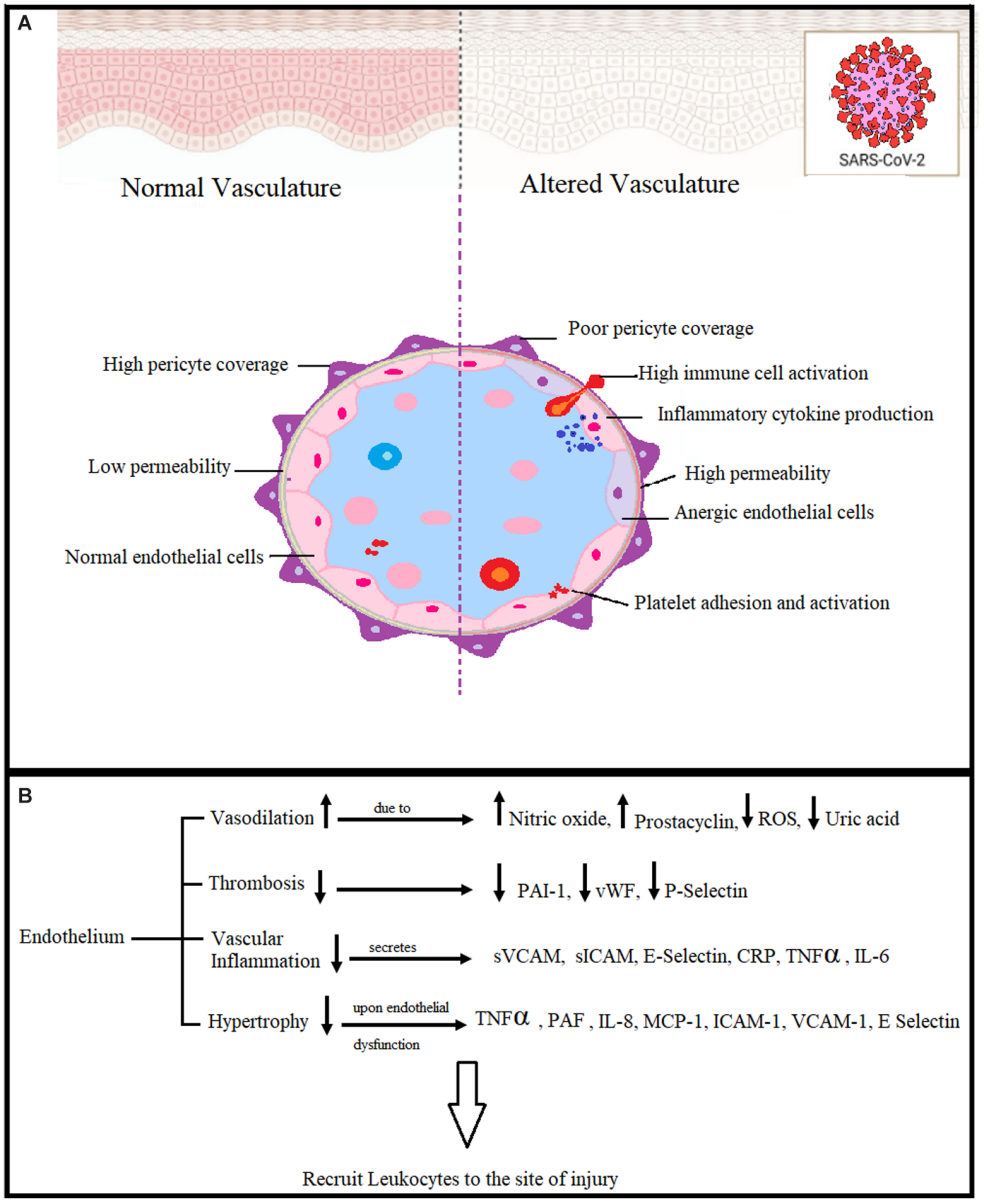
**(A)** Effects of endothelial dysfunction in COVID-19. Pericytes are BBB-supporting cells, and viral infection causes their reduction, leading to BBB breakdown and viral entry into the CNS. **(B)** The vascular endothelium is a systemic injury target. Reactive oxygen species (ROS), plasminogen activator inhibitor-1 (PAI-1), von Willebrand coagulation factor (vWF), Vascular cell adhesion molecules (sVCAM), intercellular adhesion molecules (sICAM), C-reactive protein tumor necrosis factor (TNFα), Interleukin 6 (IL-6), Platelet-activating factor (PAF), Interleukin 8 (IL-8), *Monocyte chemoattractant protein-1(MCP-1)* (19, 20).

Vascular endothelial cells show a great deal of elasticity and exhibit phenotypic heterogeneity. Their organization forms the vascular endothelium, which covers the vascular lumen as a monolayer and provides an interface between immune cells and circulating blood. They play a role in regulating vascular endothelial cells in combination with smooth muscle cells. Dysfunction of vascular endothelial cells under pathophysiological conditions can lead to disruption in vascular function (Figure 1). Kinases and GTPases regulate the barrier function of endothelial cells mediated by cell-to-cell junctions between them (21). BBB breakdown leads to many diseases of the central nervous system (CNS). The integrity of the BBB is crucial for the protection of the CNS from viral infections and other disease-causing agents, as well as for the supply of oxygen and glucose to the brain. As a result of infection, there are changes in vasculature, a reduction in pericytes that support cells for the BBB, and vascular junctions are disorganized. BBB breakdown paves the way for viral entry into the CNS, leading to inflammation, neuronal injury, and CNS diseases (22).

The most frequent pathology of COVID-19 infection is acute lung damage. Cytokine storming brought on by a viral infection may result in neurological problems, hemodynamic instability, and organ malfunction. In this condition, there are prominent systemic vascular lesions, mostly of the lungs, but also of the heart, brain, kidneys, and gastrointestinal organs. In an essay, Sardu and associates posed the question, “Is COVID-19 an endothelial disease? (23). The majority of COVID-19 infection symptoms, such as high blood pressure, thromboembolism, renal and neurological problems, and diabetes, are seen to be associated with the endothelium, making this a fascinating and crucial subject. The coronavirus over-activates the immune system, increasing inflammatory mediators and resulting in a cytokine storm. (24, 25). As a result of cytokine storming, microvessels are injured, leading to alveolar edema and pulmonary and systemic hypoxia. These events lead to respiratory distress syndrome in COVID-19 infection (26, 27).

Endothelial dysfunction is associated with respiratory distress of higher severity and COVID-19 infection. Vascular damage is initiated within microcirculation including microthrombi and capillary hemorrhages. Cytokine-induced endothelial dysfunction in the progressive stage of the disease has multi-organ implications and causes arterial hypertension, myocardial injury, diabetes, and neurological disorders (28). SP and NK-1R may be involved in cytokine storming leading to endothelial dysfunction. NEP has an indirect role in endothelial dysfunction as it is responsible for the degradation of SP under normal physiology, whereas its altered function due to any nociceptive stimuli may lead to increased SP levels in plasma and hence an enhanced cytokine storming causing endothelial dysfunctioning (2, 29, 30).

After the initiation of COVID-19 infection after a viral attack, it continues to the endothelial cells of the lungs and other organs. Endothelial dysfunction occurs mostly in the second or progressive stage of the COVID-19 pathogenesis. The STORM-2 concept proposes the biochemical mechanisms damaging the endothelium of the lung, affecting the coagulation system, vascular tone, and hemodynamic and arterial pressure regulation (5).

The virus affects the respiratory tract along with non-respiratory symptoms including cardiovascular damage. Previous studies show that most of the patients with a severe COVID-19 infection had comorbidities such as hypertension, cardiac disorders, diabetes mellitus, and obesity. SARS-CoV-2 induces cytokine storming, cellular damage, and an imbalanced renin-angiotensin system in many cell types but primarily in endothelial cells. Endothelial dysfunction induced by COVID-19 infection may cause hypoxia, myocardial injury, kidney failure, and coagulating and thrombolytic events (31).

The arteries, veins, and capillaries are lined by an inner continuous monolayer of endothelial cells. This monolayer serves as an endocrine organ and barrier between tissues and blood. Owing to its critical role in hemodynamic regulation, the endothelial cell monolayer is linked to many pathological processes (32). It is a decisive crossing point between blood and tissues. Endothelial dysfunction has been found to be associated with previous coronavirus infections (33, 34). Aging, a decline in sex hormones with age, reactive oxygen species (ROS), an increase in the ratio of circulating endothelium microparticles to progenitor cells (EMPs/PCs), and pro-inflammatory cells all contribute to endothelial dysfunction (35, 36). Damage of vascular endothelium in patients with diabetes, hypertension, renin-angiotensin imbalance, and cardiac vascular disorders may worsen COVID-19 symptoms. Thus, it is necessary to understand the mechanisms of endothelial dysfunctions in order to suggest therapeutic targets to lessen the severity of infection (37).

Hyperinflammation, hypoxia, and imbalanced RAS occur in many cell types, such as immune cells, type II alveolar cells, and endothelial cells, as a result of COVID-19 infection. High amounts of immune cell mediators cause endothelial leakage, hence, systemic inflammation and thrombosis. High levels of Angiotensin II in endothelium lead to its pro-inflammatory and pro-coagulant character. Endothelial dysfunction may result from Acute respiratory distress syndrome (ARDS)-induced hypoxia brought on by mitochondrial ROS generation, intracellular acidosis, cell signaling pathway activation, and increased blood hemodynamic resistance (38). Pneumocytes, local macrophages, and dendritic cells are the first to create chemokines and pro-inflammatory mediators in the wounded region as a response to viral infection, even though neutrophils and monocyte-macrophages are the principal producers of inflammatory mediators that cause cytokine storm. Through systemic circulation, cytokine storm spreads throughout the body and damages several organs by generating vascular leakage and coagulopathy (39).

The presence of ACE-2 in almost all organs suggests that SARS-CoV-2 may begin to spread throughout the body as soon as it enters systemic circulation. Endothelial, smooth muscle, and perivascular pericytes all have ACE-2. Significant alterations in endothelial morphology, including cell swelling, disruption to intracellular connections, and apoptosis, have been seen in post-mortem lung tissues from individuals who died with COVID-19 or ARDS (40).

The deregulation of the local RAS system brought on by the SARS-CoV-2 infection may be one molecular reason for these clinical results. The RAS system is present in organs that operate by autocrine and paracrine mechanisms without the need for circulating RAS, such as the heart, lungs, and liver (41, 42). A recent study has shown that autoantibodies targeting G protein-coupled receptors (GPCR) and RAS-related molecules are associated with disease severity in COVID-19. It was observed that out of 246 patients with COVID-19, the patients with moderate to severe symptoms had increased autoantibody levels when compared with healthy control and patients with mild symptoms. Anti-GPCR autoantibodies identified are chemokine receptor (CXR3) and RAS-related molecule AGTR1 identified as targets for antibodies with the strongest association with disease severity (43).

The organ-based RAS system, fibrogenesis pathways, and inflammation all have significant effects on the injury/repair response. In a model of acid aspiration-induced acute lung damage, mice missing ACE-2 showed considerably greater levels of pulmonary vascular permeability, which is a marker of acute lung injury/ARDS in people (44). It is hypothesized that SARS-CoV-2 binding to ACE-2 and the downregulation of ACE-2 would result in the loss of ACE-2 protective qualities in the local RAS system of the lung, regardless of the presence of an active viral infection (45).

A crucial tissue-specific RAS organ is the heart. Diminazene aceturate, an ACE-2 activator, has been shown to increase endothelial progenitor cell circulation, reduce ischemia-induced heart damage in rats, and restore the RAS system’s natural equilibrium. Lower levels of ACE-2 expression and viral RNA were found in the hearts of SARS patients after autopsies, which may help to explain the reported cardiac damage in COVID-19 cases (46). Patients with COVID-19 may be more susceptible to cardiac injuries due to ACE-2’s lack of cardioprotective activity, or patients with heart failure may be more susceptible to catching SARS-CoV-2 and experiencing associated cardiac damage (47). These findings imply that SARS-CoV-2 may offer a variety of risks to the cardiovascular system, as well as the pulmonary and cardiac vasculature, via altering ACE-2 function. Although mechanistic research is required in this situation to identify high-risk patients and create viable therapeutics, other routes through the circulatory system and other target organs are also required (45).

### Ace-2 the receptor for coronavirus

The renin-angiotensin system, which is connected to the regulation of the heart and blood vessels, is where the angiotensin-converting enzyme (ACE) plays a major role. V N Orekhovich discovered it for the first time at Moscow’s Institute of Biological and Medicinal Chemistry of the Russian Academy of Medical Sciences in 1963 (48). The renin-angiotensin system is involved in the synthesis of the pro-hypertensive peptide angiotensin II (ANG-1-8) as well as the hydrolysis of the kinin system byproduct bradykinin. A specific peptide hydrolase having a systemic role is angiotensin convertase (49). ACE immunolocalization on the luminal surface of lung endothelial cells was identified (50). The discovery of coupled regulators of the blood and vascular system, such as nitric oxide, prostaglandins, endothelin, and prostacyclins, which have varied consequences in illnesses, further emphasized the therapeutic significance of ACE research. Increased ACE activity is caused by AngII and functional polymorphisms in the ACE gene, which increases vulnerability to asthma, pulmonary hypertension, and chronic obstructive pulmonary disease (COPD). ARDS is characterized by increased ACE-2 expression, which is essential and protective (7).

Without the “kallikrein” enzyme, which regulates blood pressure, ACE as kinase II is insufficient. Hageman factor, kallikrein, kininogen, and bradykinins operate as counterbalances to ACE and angiotensin II binding the receptors, which cause physiological consequences, in physiological and pathological processes. These substances have an impact on the lungs’ endothelium, which controls hemodynamic equilibrium (5).

The large angiotensin polypeptide and its various fragments, as well as bradykinin, are processed by ACE-2, which was first identified in 2000 (51, 52). It was later discovered that ACE and ACE-2 have similar catalytic domains and both are involved in the processing of these compounds. However, bradykinin or neurotensin cannot be hydrolyzed by ACE-2. Due to its significance as a primary contributor to COVID-19 infection, ACE2 is now attracting attention. Coincidentally, the plasma membrane of host cells includes ACE2, which SARS-CoV-2 may bind to. Compared to the original viral strain, SARS-CoV, SARS-CoV-2 has a ten to twenty-fold greater binding affinity (53). The ACE-2 receptor is used by the SARS-CoV-2 coronavirus to enter host cells (54). SARS-CoV-2 is mostly found in the alveolar epithelial cells of the lungs, even though ACE-2 is damaged in numerous organs (55).

ARDS, hypertension, and other pathogenic processes are all regulated by ACE-2. As type 2 diabetes and hypertension worsen, ACE-2 activity and blood pressure both decline. Thus ACE-2 is targeted in many treatments for controlling diabetes including ACE inhibitors medications, endogenous ACE-2 activators, ACE-2 gene therapies, human recombinant ACE-2, and Ang-II receptor blockers. ACE-2 is also a receptor of SARS-CoV-2 and facilitates the entry of the virus inside the host cell. Medications used by clinicians for the treatment of COVID-19 are classified into two classes: one targets the immune system and the other targets the interaction of ACE-2 with SARS-CoV-2 (56).

**Figure 2:**
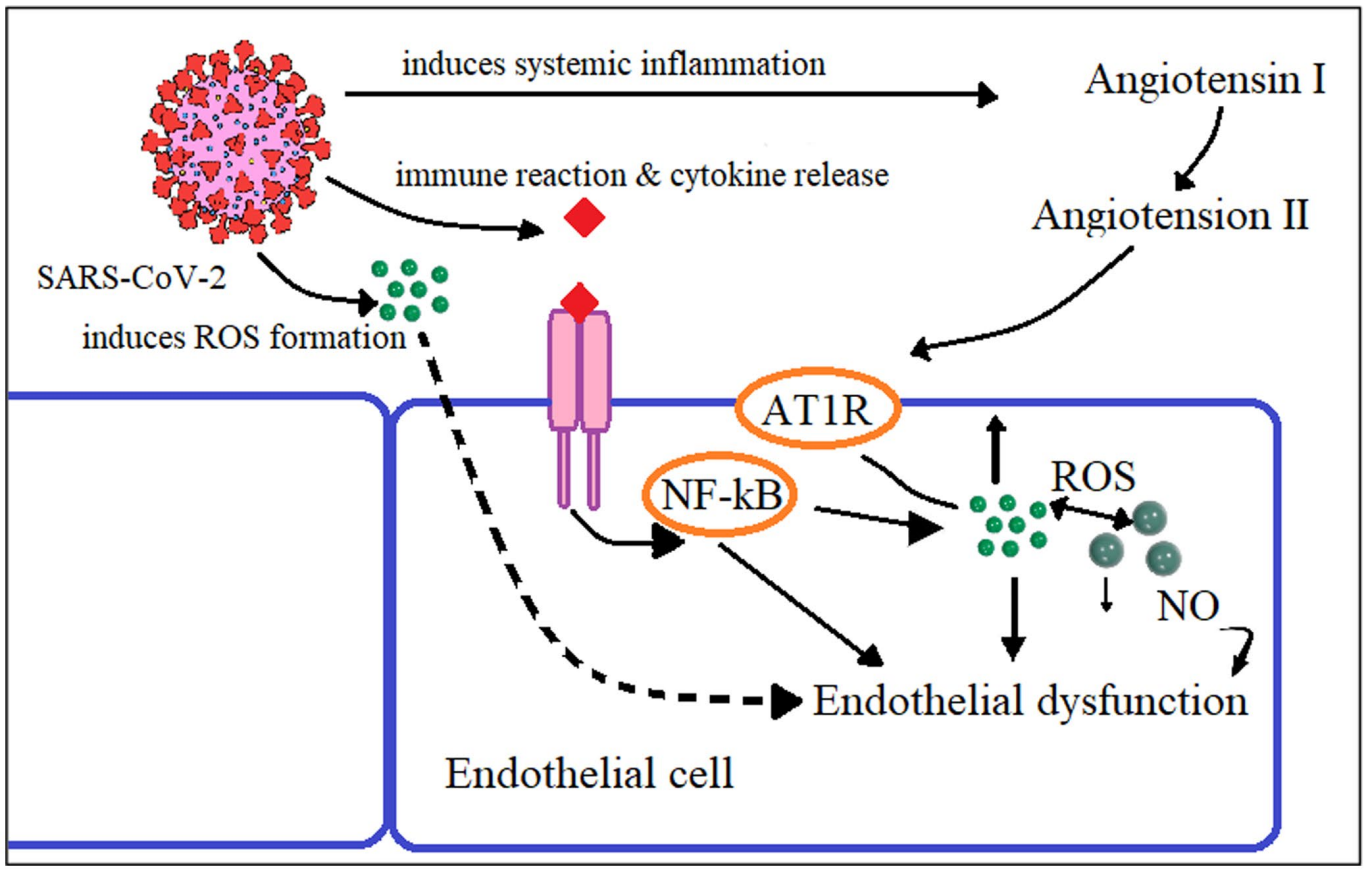
Proposed mechanism of endothelial dysfunction in SARS-CoV-2 infection Reactive oxygen species (ROS), Angiotensin-2 Type I Receptor (AT1R), Nuclear factor Kappa B (NF-κB), Nitric oxide (NO).

Localization of ACE-2 in the human endothelium of arterial and venous vessels and in the arteries of smooth muscles of almost all organs is established. The ACE-2 receptors in the mucous membranes of the nose, mouth, stomach, and intestines are the sites for viral invasions (40). When SARS-CoV-2 binds to the ACE-2 receptor, hyperinflammation and the start of a cytokine storm occur. The inhibition of ACE-2 receptors by the coronavirus causes an imbalance in the ratio of proinflammatory mediator expression (Figure 2). When ACE-2 is blocked by a coronavirus, ACE expression—in this instance, kininase II expression—increases. As a result, the beneficial effects of bradykinin on cells are reduced, and vice versa, and its amount, a proinflammatory substance that affects the pulmonary epithelium, rises. Increased neutrophil activity and COVID-19 severity are results of kinin cytotoxicity (57). Cytokines, such as IL-1B and TNF-α, activate the kinin receptor BKB1R, and the receptor blockade can be a therapeutic strategy for acute respiratory distress (58).

### Role of SP and fragments in the destruction and recovery of the brain

In our previous studies, we have explored the role of Substance P (SP) in the pathogenesis of COVID-19 infection by initiating cytokine storming (1, 2). SP is a neuropeptide and neurohormone, specifically released from the trigeminal ganglion, and is associated with nociception and inflammation, apart from normal physiological functions (4). Its release is triggered as a consequence of nociception and induces inflammation and endothelial dysfunction. Inflammatory mediators are released in blood vessels, causing bronchoconstriction, respiratory distress, and thrombosis. It directly affects the cardiorespiratory control, sleep–wake cycle, and respiratory regulation (2). A phenomenon of latency has also been proposed in patients with COVID-19, in one of our previous studies (1).

### Therapeutic and preventive concepts

Treatments such as renin-angiotensin system inhibitors, beta-blockers, and statins may improve endothelial function and other related complications. Additionally, we suggest a novel therapeutic strategy, i.e., Neurokinin-1 Receptor inhibitor along with dexamethasone, which is a glucocorticoid, for the prevention and treatment of COVID-19 infection (11). A clinical trial performed in our previous study has shown very promising results (Mehboob R).

### Perspectives for the future

Multiorgan clinical symptoms and several post-COVID signs are seen in individuals with COVID-19 (59, 60). Patients with pre-existing comorbidities, such as hypertension, obesity, diabetes, or cardiovascular disease, have endothelial dysfunction that seems to play a significant role in the etiology of COVID-19 (61–63).

## Conclusion

In this study, we explored how COVID-19-related endothelial dysfunction might decrease organ perfusion and lead to thromboembolic events such as acute myocardial infarction, renal failure, and pro-coagulant states. Endothelial dysfunction may contribute to the pathogenesis of COVID-19, particularly in patients with comorbidities such as hypertension, diabetes, cardiac disorders, etc. The balance between ACE and its homolog, ACE-2, is essential for regulating AngII levels. Any changes to the ACE/ACE-2 ratios and cytokine stress are linked to the endothelium system becoming dysfunctional and may lead to vascular diseases.

These new understandings of the COVID-19 molecular processes may enhance patient care and therapy and offer fresh hope on how to deal with the pandemic (31).

*Virchow was not only the founder of cellular pathology and the Virchow’sche Trias. He also said that we must not only look for single cells. We must keep the **homöostasis** (in 1) between cells. This homöostasis principle must also be the basis for preventive measurements against corona infection.*

## Data availability statement

The original contributions presented in the study are included in the article/Supplementary material, further inquiries can be directed to the corresponding author.

## Author contributions

RM and JPvK have contributed in the conceptualization and design of study and also contributed in writeup. KE, MA, and AB have contributed in writeup and finalization. All the authors have read and approved the final manuscript.

## Conflict of interest

The authors declare that the research was conducted in the absence of any commercial or financial relationships that could be construed as a potential conflict of interest.

## Publisher’s note

All claims expressed in this article are solely those of the authors and do not necessarily represent those of their affiliated organizations, or those of the publisher, the editors and the reviewers. Any product that may be evaluated in this article, or claim that may be made by its manufacturer, is not guaranteed or endorsed by the publisher.
